# DNA sequence analysis of keratinophilic fungi isolated from livestock stables in the East of Iran

**DOI:** 10.18502/cmm.6.3.3981

**Published:** 2020-09

**Authors:** Zohre Behzadi, Abdol majid Fata, Saeed Parham, Hossein Zarrinfar, Mohammad Javad Najafzadeh

**Affiliations:** 1 Department of Parasitology and Mycology, Faculty of Medicine, Mashhad University of Medical Sciences, Mashhad, Iran; 2 Cutaneous Leishmaniasis Research Center, Mashhad University of Medical Sciences, Mashhad, Iran; 3 Cancer Molecular Pathology Research Center, Mashhad University of Medical Sciences, Mashhad, Iran

**Keywords:** Iran, Keratinophilic fungi, Livestock, Qayen

## Abstract

**Background and Purpose:**

Keratinophilic fungi play an important role in the decomposition of keratinous substances in nature. This capacity induces dermatomycosis in both humans and livestock. The soil of livestock stables can be a reservoir of keratinophilic fungi. Therefore, the present study was conducted to isolate and identify keratinophilic fungi in the soil of the livestock stables located in Qayen, South Khorasan Province, Iran.

**Materials and Methods:**

This study was conducted on 62 soil samples collected from livestock stables. The samples were cultured by means of hair bait technique (HBT). The identification of the isolates was performed based on their morphological characteristics and then confirmed by polymerase chain reaction and sequencing of the ITS regions of ribosomal DNA.

**Results:**

A total of 118 isolates of 7 species from 5 genera were identified. *Aphanoascus verrucosu* (n=70, 59.36%) was detected as the dominant keratinophilic fungus, followed by *Arthroderma quadrifidum* (n=13, 11.01%), *A. terreus* (n=12, 10.16%), *Acremonium* (n=12, 10.16%), *A. gertleri* (n=5, 4.23%), *Fusarium equiseti* (n=3, 2.54%), and *Uncinocarpus reesii* (n=3, 2.54%).

**Conclusion:**

Different keratinophilic fungi were isolated from the soil of livestock stables; however, *A. verrucosu* was found to be the dominant species.

## Introduction

The keratinophilic fungi are a group of dermatophyte and non-dermatophyte molds. Most of these fungi inhabit the soil; however, some of them are plant pathogens.

Furthermore, dermatophytes have humans, animals, and soils as ecological niche [ 1 ]. Dermatophytes account for a number of skin, hair, and nail lesions, named as dermatophytosis (ringworm or tinea). However, some other keratinophilic species can cause similar infections [ 2 , 3 ]. During the last decades, numerous papers have been published on dermatomycosis caused by non- dermatophytic filamentous fungi [ 4 ]. These studies have been indicative of the importance of other keratinophilic fungi in the development of dermatomycosis.

Keratin is a protein unit, which participates in the structure of the hair, skin, feather, and nails. The decomposition of keratin by microorganisms results in the production of hydrogen sulfide and ammonia, which are toxic compounds and can cause problems for humans and animals. On the other hand, the degradation of keratin by keratinophilic fungi leads to the conversion of this structural protein to their constituent elements (e.g., carbon, hydrogen, and sulfur) and their return to the natural cycle.

Keratinophilic fungi secrete proteinase enzyme, which plays an important role in food, textile, and detergent industries. However, this enzyme is harmful to animal products, such as wool and skin [ 3 ].

Since the soil is regarded as a reservoir of keratinophilic species, the identification of these fungi in soil is a matter of fundamental importance. Studies on soil keratinophilic fungi have been conducted in various parts of Iran (e.g., Khorasan [ 11 ], Shiraz [ 3 ], Sari [ 12 ], Qazvin [ 13 ], and Ahwaz [ 14 ]) and around the world (e.g. Egypt [ 5 ], Australia [ 6 ], Libya [ 7 ], Bahrain [ 8 ], Brazil [ 9 ], and Korea [ 10 ]).

Since the soil of livestock stables contains keratinous substances, the isolation of keratinophilic fungi from these locations is expected. Regarding this, the present study was conducted to determine the spectrum of fungal keratinophilic species in the soil of some livestock stables in Qayen, South Khorasan, Iran.

## Materials and Methods

This cross-sectional descriptive study was conducted on 62 soil samples collected from 45 various livestock stables located in Qayen, from May to December of 2017. For sample collection, 300-400 g soil was taken from a depth of 3-5 cm of the soil using a disposable spoon and then transferred to the laboratory in labeled polyethylene bags. The isolation was performed by the Vanbreuseghem's hair baiting technique (HBT) [ 2 ]. To this end, the soil sample was placed in a sterile 90-mm Petri dish with a few sterile hair strands of an immature girl on the surface of the soil and then moistened with sterile distilled water. The Petri dishes were incubated at 25-28°C for 2 months [ 2 ]. When a colony appeared on the hair, it was subcultured on Sabouraud's dextrose agar with chloramphenicol (Conda, Spain). The colonies were identified based on their microscopic characteristics [ 4 ]. Thereafter, among the similar isolates in each petri dish, one colony was selected for molecular identification.

DNA was extracted using the Dena Zist DNA extraction kit (Dena Zist, Iran) according to the manufacturer’s instructions. The DNA extracts were stored at -20°C until used. Ribosomal DNA internal transcribed spacer (ITS) regions were amplified and sequenced using primers ITS1 (5’ TCCGTAGG TGAACCTGCGG 3’) and ITS4 (5’ TCCTCCGC

TTATTGATATGC 3’) [ 15 ]. Polymerase chain reaction (PCR) was also performed in a reaction mixture, containing 12.5 µl 2X Master Mix Red (Ampliqon, Denmark), 1 µl of each primer (10 pmol), 1 µl DNA, and enough molecular grade water at a final volume of 25 µl. In addition, amplification was carried out in an ABI PRISM 2720 (Applied Biosystems, Foster City, USA) thermo-cycler. The PCR condition included 95°C for 2 min, followed by 35 cycles at 95°C for 35 sec, 52°C for 30 sec, and 72°C for 1 min, as well as a delay at 72°C for 7 min.

The concentrations of amplicons were estimated on 1.5% agaros gel, photographed, and analyzed by the Gel Doc XR system (Biorad), with SmartLadder (Eurogentec, Seraing, Belgium) as a size and concentration marker. The amplicons were then subjected to direct sequencing using the ABI prism BigDyeTM terminator cycle sequencing kit (Applied Biosystems, Foster City, USA) and analyzed on an ABI Prism 3730XL Sequencer. The sequences were analyzed in Seqman software (DNASTAR, Wisconsin, USA), and for identification, they were BLASTed against those presented in the dermatophyte database of Westerdijk Institute (www.westerdijkinstitute.nl) and GenBank [ 16 ].

## Results and discussion

In this study, 118 colonies belonging to 5 genera and 7 species were detected in 62 samples. *Aphanoascus verrucosa* was identified as the dominant keratinophilic species (n=70, 59.36%). The other most common fungi detected in this study included *Arthroderma quadrifidum*, (n=13, 11.01%), *A. terreus* (n=12, 10.16%), *Acremonium* species (n=12, 10.16%), *A. gertleri* (n=5, 4.23%), *Fusarium equisetai* (n=3, 2.54%), and *Uncinocarpus reesii* (n=3, 2.54%). Table 1 shows the prevalence of keratinophilic speciens in the soil samples based on the isolated location.

**Table 1:** Number of isolated species based on each sampling area in the soil of Qayen city and frequency of colonies with respect to species

Species	Collected area (rural/urban)	Number of colony collected (%)
*Aphanoascus verrucosu*	Shahrakht (rural)	20 (16.9%)
	Ostrich stable (urban)	50 (42.46%)
*Trichophyton terrestre (Arthroderma quadrifidum)*	Pishbar-Garmab (rural)	13 (11.01%)
*A. terreus*	Ghomenja-Tighdar Pishbar- Mehdi Abad (rural)	12 (10.16%)
*Acremonium sp.*	Haji Abad, KHezrii (rural)	12 (10.16%)
*T. vanbreuseghemii (A. gertleri)*	Khorvaj (rural)	5 (4.23%)
*Fusarium equisetai*	Ostrich Stable (urban)	3 (2.54%)
*Uncinocarpus reesii*	Bashiran (rural)	3 (2.54%)
Total	16	118 (100%)

In our study, 65 colonies were isolated from rural areas, while 53 colonies were recovered to the urban areas. The urban species belonged to the two genera of *Fusarium* and *Aphanoascus*, and other isolates were from rural areas. Among the investigated rural areas, Shahrakht (i.e., a town located in Southern Khorasan) had the highest number of colonies. Furthermore, regarding different stables in the urban areas, the highest number of colonies was detected in the soil samples obtained from ostrich stables.

Keratinophilic fungi play an important role in the decomposition of keratin in nature. A number of keratinophilic fungi like dermatophytes can cause problems for humans and livestock with high keratinolitic power. The soil of the livening place of the livestock can contain the spores of keratinophilic fungi and potentially act as dermatomycosis agents. *Trichophyton terrestre (A. quadrifidum)* was the mostly isolated dermatophyte species. This is in line with the results obtained by Pontes et al. [ 9 ] reporting *T. terrestre* as the most frequent dermatophyte isolated from Brazil soil. Pontes et al. identified 101 isolates of 8 different dermatophytes, whereas in the current study, two dermatophytespecies were isolated. This could be due to the difference in the climatic condition of the two regions; in this regard, while one region is warm and humid, the other one is arid and semidesert.

In addition, our result was close to those reported by Rezvana et al., presenting *T. terrestre* as the second most frequently isolated dermatophyte after *Microsporum gypsum* in the soil of Riyadh, Saudi Arabia [ 17 ]. Similarly, Shookohi et al. introduced *T. terrestre* as the second dermatophyte after *T. ajeloi (A. uncinatum)* in Sari, Iran [ 12 ]. All these reports indicate the widespread presence of this dermatophyte in different climates.

*A. gertleri* was the second most frequently isolated dermatophyte. There has been no report regarding the presence of this species in any soil studies conducted in Iran so far, except in a case of tinea capitis studied in Mashhad based on morphologic findings [ 18 ]. Regarding this, the present study reported the first confirmed case of this rare dermatophyte isolated from Iran. *Aphanoascus* is an ascomycetous mold with keratinophilic activity. It has 12 species and is not a dermatophyte fungus; moreover, it does not respond to antifungal drugs. *Aphanoascus* is highly similar to *Chrysosporium* and has been reported in onychomycosis infections [ 19 - 21 ].

In this study, *Aphanoascus* was the most frequently isolated keratinophilic fungi. This result is similar to those obtained by Deshmukh et al., who introduced *Aphanoascus* genus as the keratinophilic fungi most frequently isolated from Bahrain soil [ 8 ]; however, their species were not the same as those identified in the present research. The species detected in the current study were *A. verrucosa* and *A. terreus*; however, those reported by Deshmukh et al. included *A. fulvuscence* and *A. punsolae*. In addition, *Aphanoascus* was identified as the most frequent genus in Ahwaz soil (south of Iran) by Rezaei Matehkolaei et al. [ 14 ]. This similarity in the results obtained in Bahrain, Ahwaz, and east of Iran could be due to relative similarity in moisture and dry air.

In this study, *Acremonium* prevalence was in the third place, which is in line with the results presented by Pakshir et al. in Shiraz, Iran [ 3 ]. However, in some studies like those performed by Aghamirian et al. in Qazvin [ 13 ], Shokohi et al. in Sari (north of Iran) [ 12 ], and Rezvana et al. in Riyadh [ 17 ], this species was the most frequent keratinophilic fungus. *Fusarium* is a ubiquitous fungal species found in plants, soil, and water [ 22 ]. *Fusarium* can cause localized infection in healthy individuals, especially in those with compromised immune systems. In this regard, 15 species of this genus are able to cause such diseases as keratomycosis, mycetoma, and onychomycosis in humans [ 23 , 24 ]. There are many cases of onychomycosis caused by *Fusarium* species in Iran [ 25 ].

Prior to this study, Moalaei et al., investigating the soil of Southern and Razavi Khorasan [ 11 ], identified *Fusarium* species as the most isolated keratinophilic fungi from Qayen soil. However, no dermatophytes were isolated in the mentioned study. Nonetheless, in the present study, the most frequently isolated keratinophilic fungi were *Aphanoascus* species; in addition, two dermatophytes species were isolated. This difference between the results of the two studies performed on the same region can be due to the differences in the type of study population and sample size. In another study, Moalaei et al. collected 10 soil samples from the agricultural area of this city, while in the present study, 62 samples were taken from the livestock living place.

In this study, *A. verrucosa* was the most frequent keratinophilic fungus. Furthermore, *A. gertleri* and *T. terrestre* were isolated as two dermatophytes species. Based on the results of the present research, the soil of livestock living areas can be considered a source of different keratinophilic fungi, especially dermatophytes. Therefore, caution is advised when working in these areas.

## Conclusion

Various keratinophilic fungi were isolated from the soil of livestock stables located in Qayen, South Khorasan Province, Iran. *A. verrucosu* was found to be the dominant species among the keratinophilic fungi.
